# The identification of novel loci required for appropriate nodule development in *Medicago truncatula*

**DOI:** 10.1186/1471-2229-13-157

**Published:** 2013-10-11

**Authors:** Agota Domonkos, Beatrix Horvath, John F Marsh, Gabor Halasz, Ferhan Ayaydin, Giles ED Oldroyd, Peter Kalo

**Affiliations:** 1 Agricultural Biotechnology Center, Gödöllő 2100, Hungary; 2 John Innes Centre, Norwich NR4 7UH, UK; 3 Cellular Imaging Laboratory, Biological Research Center, Szeged 6726, Hungary

**Keywords:** *Medicago truncatula*, Legume, Symbiosis, Mutant screen, Ineffective nitrogen fixation mutant

## Abstract

**Background:**

The formation of functional symbiotic nodules is the result of a coordinated developmental program between legumes and rhizobial bacteria. Genetic analyses in legumes have been used to dissect the signaling processes required for establishing the legume-rhizobial endosymbiotic association. Compared to the early events of the symbiotic interaction, less attention has been paid to plant loci required for rhizobial colonization and the functioning of the nodule. Here we describe the identification and characterization of a number of new genetic loci in *Medicago truncatula* that are required for the development of effective nitrogen fixing nodules.

**Results:**

Approximately 38,000 EMS and fast neutron mutagenized *Medicago truncatula* seedlings were screened for defects in symbiotic nitrogen fixation. Mutant plants impaired in nodule development and efficient nitrogen fixation were selected for further genetic and phenotypic analysis. Nine mutants completely lacking in nodule formation (Nod-) represented six complementation groups of which two novel loci have been identified. Eight mutants with ineffective nodules (Fix-) represented seven complementation groups, out of which five were new monogenic loci. The Fix- *M. truncatula* mutants showed symptoms of nitrogen deficiency and developed small white nodules. Microscopic analysis of Fix- nodules revealed that the mutants have defects in the release of rhizobia from infection threads, differentiation of rhizobia and maintenance of persistence of bacteria in nodule cells. Additionally, we monitored the transcriptional activity of symbiosis specific genes to define what transcriptional stage of the symbiotic process is blocked in each of the Fix- mutants. Based on the phenotypic and gene expression analysis a functional hierarchy of the *FIX* genes is proposed.

**Conclusions:**

The new symbiotic loci of *M. truncatula* isolated in this study provide the foundation for further characterization of the mechanisms underpinning nodulation, in particular the later stages associated with bacterial release and nodule function.

## Background

Legumes have the ability to develop root endosymbioses with soil bacteria, termed rhizobia. This association helps the plant in the capture of nitrogen and as a result legumes can grow in low nutrient soil and are excellent sources of plant protein and indispensable crops for sustainable agriculture [1]. Rhizobial bacteria modulate the growth and development of the plant to facilitate colonization and the establishment of the symbiotic interaction (for review, see [2]).

The legume-rhizobial interaction commences following mutual recognition of host and rhizobial signaling molecules. Plant recognition of Nodulation (Nod) factors produced by the rhizobial partner activates many of the developmental processes in the plant associated with nodule formation [3]. The attachment of rhizobia on root hairs, in conjunction with production of Nod factor, induces the formation of a tube-like structure called an infection thread (IT), which extends through the root epidermis towards the developing nodule primordia. Bacteria are released from the ITs into the cytoplasm of nodule cells [2], with the bacteria surrounded by a plant derived membrane, creating a new subcellular compartment called symbiosome. Bacteria in the symbiosomes divide and differentiate into their symbiotic forms [4], termed bacteroids. Within the root nodule the plant partner supplies the bacteroids with photosynthetic products in exchange for ammonia converted from atmospheric nitrogen.

The mature *M. truncatula* nodule displays a developmental gradient of cells creating zones typical of indeterminate-type nodules [5]. A persistent meristematic region (zone I) at the nodule apex ensures continual growth and development of the nodule. Bacteria colonize the nodule in ramifying ITs within the infection zone (zone II) and bacterial release and differentiation in plant cells begins within this region. The differentiation of both plant cells and bacteroids are completed in the few cell layers of the interzone (zone II-III). The major part of the mature nodule is composed of the symbiotic zone (zone III) wherein nitrogen fixation takes place. The basal part of older nodules contains a senescence region (zone IV) wherein bacteroids and nodule cells undergo degradation.

The development of the model legumes, *Medicago truncatula* and *Lotus japonicus* and their substantially completed genome sequences [6,7] has greatly advanced molecular studies of legume symbiotic associations. This genetic dissection in the model legumes has identified many genes required for nodulation: components of the symbiotic signaling (Sym) pathway (recently reviewed by [8-12]), genes functioning in regulation of nodule number [13-16], bacterial infection [17-19], bacterial differentiation [20], maintenance [21,22] and nutrient transport [23,24]. In this study, we have attempted to further dissect the processes associated with nodule development, with a particular focus on the later stages of bacterial infection and nodule maturation. Fast neutron bombarded and EMS mutagenized *M. truncatula* populations were screened for nodulation mutants. The non-nodulating (Nod-) and non-nitrogen-fixing (Fix-) mutants were selected from the candidate mutants and characterized further in this study. Allelism tests with known loci revealed a number of new complementation groups, defining new genes required for both the early and late stages of nodule development. Microscopic analyses, coupled with gene expression studies in the Fix- mutants revealed genetic loci required at specific stages of nodule development.

## Results

### Identification of new nodulation-defective *M. truncatula* mutants

In order to identify additional genetic loci required for symbiotic nitrogen fixation, a large-scale forward genetic screen was undertaken with fast neutron bombarded and ethyl methane sulfonate (EMS) mutagenized *M. truncatula* jemalong populations. The frequency of chlorophyll deficient (albino) phenotypes among the M2 plants was 2.6% indicating the success of the mutagenesis [25]. Approximately 38,000 seedlings of about 600 M2 families [26] were grown in media of low N content and screened for symbiotic phenotypes five-six weeks post inoculation with wild type *Sinorhizobium meliloti* strain B1. Plants were screened for nodule defects, with potential mutants lacking nodules and those carrying defective nodules being kept for secondary screening. The progeny of the putative mutants were subjected to a second round of tests to confirm their symbiotic phenotypes and finally nine nodulation-defective (Nod-) and numerous ineffective nodulation (Fix-) mutants were selected for further analysis.

The Nod- category included mutants that did not form nodules at all, plants developing small bumps or those showing greatly reduced nodulation. Root hair deformation assays and infection with *S. meliloti* was carried out on these mutants to analyze the response of root hairs to bacterial Nod factor (NF) and the induction of cortical cell division following rhizobial inoculation. Based on the early symbiotic responses Nod- mutants were classified and crossed to known Nod- mutants to reveal allelic relationships (Additional file 1A). The allelism tests identified new alleles of *dmi2* and *nsp2* and three new alleles of *dmi3*. Subsequent sequence analysis of the *dmi2* allele (*dmi2-5*) revealed a 97 bp deletion in the extracellular domain of *DMI2*, while the *dmi3* mutants revealed identical 7 bp deletions (indicating that these mutant lines represented siblings) and thenceforward they will be designated as a single novel *dmi3* allele (*dmi3-3*). The 7 bp deletion in *dmi3-3* starts at position 771 of the coding sequence and generates two immediate consecutive stop codons. Two additional nodulation-defective mutants representing new alleles of *LIN* were also isolated in this screen and these have been described in [18]. In addition one allele of *NIN* was identified that was described by [26] and a new complementation group *STA*, that develops reduced numbers of nodules, was described in [27].

### Characterization of new loci involved in nodule development and bacterial infection

**Figure 1:**
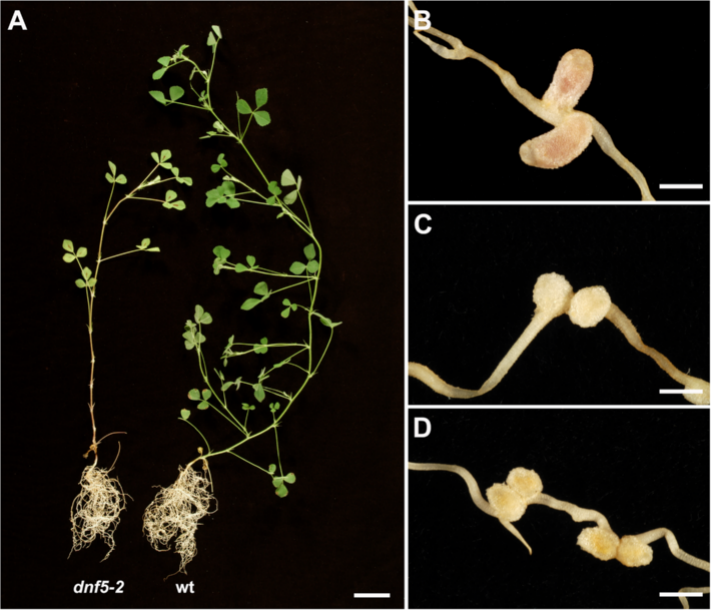
**The gross phenotypes of nodulation defective mutants.** The nodulation phenotype of the Fix- mutant *dnf5-2* as compared to wild-type *M. truncatula* 4 weeks postinoculation (wpi) with *S. meliloti* 1021. Ineffective mutants, such as *dnf5-2* display retarded growth and show other symptoms of nitrogen deficiency, such as leaf yellowing **(A)**. Wild-type plants developed pink cylindrical nodules **(B)**. Spherical or slightly elongated white nodules were found on ineffective mutant roots. **(C)**, *dnf5-2*; **(D)**, 13U mutant. Scale bars: 2 cm in **A**, 1 mm in **B** to **D**.

**Figure 2:**
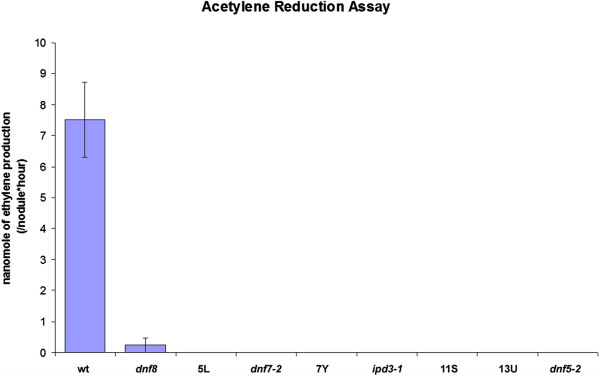
**The nitrogenase activity measured by acetylene reduction.** Nitrogenase activity was absent in all the Fix- mutants except *dnf8*, where it was greatly reduced. Three to six nodules were pooled from each *M. truncatula* line in three independent experiments. Error bars represent SE.

The Fix- mutants were defined by symptoms of nitrogen starvation under symbiotic conditions and the development of small white nodules (Figure 1). Supplementation with combined nitrogen restored normal growth habits in eight of the Fix- mutants, indicating specific defects in the ability to establish appropriate conditions for nitrogen fixation. This was further validated using acetylene reduction that measures the activity of bacterial nitrogenase [28]. After 18 days post inoculation with *S. meliloti* wild-type nodules displayed high levels of acetylene, but no acetylene reduction was detected on any of the 8 backcrossed Fix- mutants, except one mutant, *dnf8* (see below), in which a very low rate of nitrogen fixation was observed (Figure 2).

The F2 segregation ratios of backcrossed mutants indicated that all 8 Fix- mutants segregated as single recessive loci (Additional file 2). To assess allelism the Fix- mutants were crossed to each other and to the previously identified *dnf* mutants [29]. This revealed alleles of *dnf5* and *dnf7* (*dnf5-2* and *dnf7-2*) and one novel complementation group that we will refer to as *dnf8* (Additional file 1B). A mutant allele of *IPD3* was identified and this allele has been described in [30].

Four additional Fix- mutants were partially analysed. The allelism tests revealed that 5L and 11S had mutations at the same locus, but incomplete analyses of these along with 7Y and 13U meant that we were not able to define with allelism tests alone whether these represent new genetic loci, or alleles of already defined *DNF* loci. However, the allelism tests performed to date reveal no similarities with the *DNF* loci tested (Additional file 1B). Rather than complete the allelism tests we decided to undertake preliminary genetic mapping to ascertain whether these four mutants mapped to the sites of known loci. The comparison of the genetic map positions of these 3 loci (Additional file 3) and the previously identified *dnf* mutants (D. Wang, personal communication) suggested that either the mutant loci 5L/11S, 7Y and 13U are mapped to genomic locations with unknown symbiosis genes or the known symbiotic locus in the corresponding region was not allelic (13U and *dnf2*; [22]). The genetic mapping data and mutant phenotypes described below suggested that mutants 5L/11S, 7Y and 13U represent novel loci.

### Ineffective mutants show arrests in different stages of symbiotic nitrogen fixation

**Figure 3:**
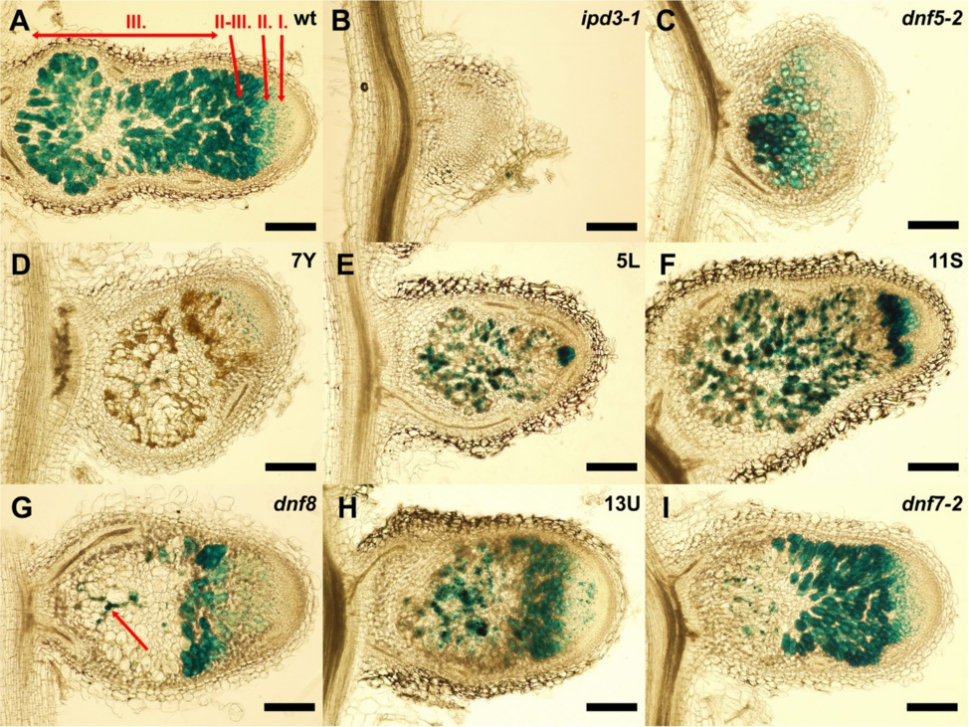
**The degree of rhizobial infection in Fix- mutants.** Nodule sections of wild-type **(A)** and Fix- mutants **(B**-**I)**. Nodules were harvested 3 weeks post inoculation with *S. meliloti* 1021 expressing the *lacZ* gene. 70 μm thick nodule sections were stained with X-gal to detect β-galactosidase activity. Nodules of **(B) ***ipd3-1*, **(C)***dnf5-2*, **(D)** 7Y, **(E)** 5L, **(F)** 11S, **(G)***dnf8*, **(H)** 13U and **(I) ***dnf7-2* mutants show various degrees of bacterial colonization. Roman letters on wild-type nodules **(A)** indicate the zones of a mature nodule: I., meristem; II., infection zone; II-III., interzone; III., nitrogen-fixation zone. The arrow shows an infection thread in panel **G**. Bars represent 200 μm.

In order to assess the stage at which the defects occurred in the Fix- mutants, we inoculated with *S. meliloti* 1021 (pXLGD4) which constitutively expresses the *LacZ* reporter gene (*hemA::lacZ*; [31]). The Fix- mutants developed exclusively white spherical or slightly cylindrical nodules except *dnf8* on which an occasional pale pink nodule could be observed. To visualize the presence of bacteria in the nodules we stained longitudinal sections of 21-day-old nodules following assays for β-galactosidase activity. The extent of bacterial colonization in the nodule zones was examined by light microscopy (Figure 3A-I). Wild type nodules showed typical zonation [5] (Figure 3A) and no such zonation was observed in nodules of *ipd3-1* and *dnf5-2* (Figure 3B and C). The majority of the nodules formed on *ipd3-1* roots were spherical with abnormal nodule apices (Figure 3B), but a small number of nodules developed into elongated cylindrical structures [30]. Neither class of *ipd3-1* nodules contained cells with released bacteria, indicating the essential function of *IPD3* for bacterial release. In contrast to *ipd3-1*, the *dnf5-2* and *dnf7-2* nodules had cells containing bacteria (Figure 3C and I), but no characteristic zonation of the indeterminate nodules was observed in *dnf5-2* nodules (Figure 3C) and the nitrogen-fixation zone was devoid of rhizobia except a few sporadic infection threads (Figure 3I) in *dnf7-2* nodules.

The other Fix- mutants developed more or less elongated nodules (Figure 3D-I), but the nodule zonation or occupancy by rhizobia was impaired in these mutants. The nodules of 7Y showed extremely low bacterial occupancy; bacteria could be detected only in a few cells of the infection zone and only a few infection threads were present in the nitrogen fixation zone (Figure 3D). Moreover an extensive brown pigmentation was observed throughout the nodule. Sporadic brown pigmentation was also present in 5L and 11S nodules (Figure 3E and F) and to a lesser degree in 13U nodules (Figure 3H). We believe that this brown pigmentation may be associated with the senescence of cells within the nodules of these Fix- mutants. 5L and 11S mutants developed nodules with a narrow infection zone containing several infected cells, but infected cells in the intermediate and nitrogen fixation zone appeared to degrade (Figure 3E and F). In *dnf8* nodules some infected cells occurred in the interzone, but no infected cells and only a few infection threads were found in the nitrogen fixation zone (Figure 3G). Similarly the 13U nodules showed bacteria within the cells of the infection zone, but low levels of infection within the nitrogen fixation zone (Figure 3H).

**Figure 4:**
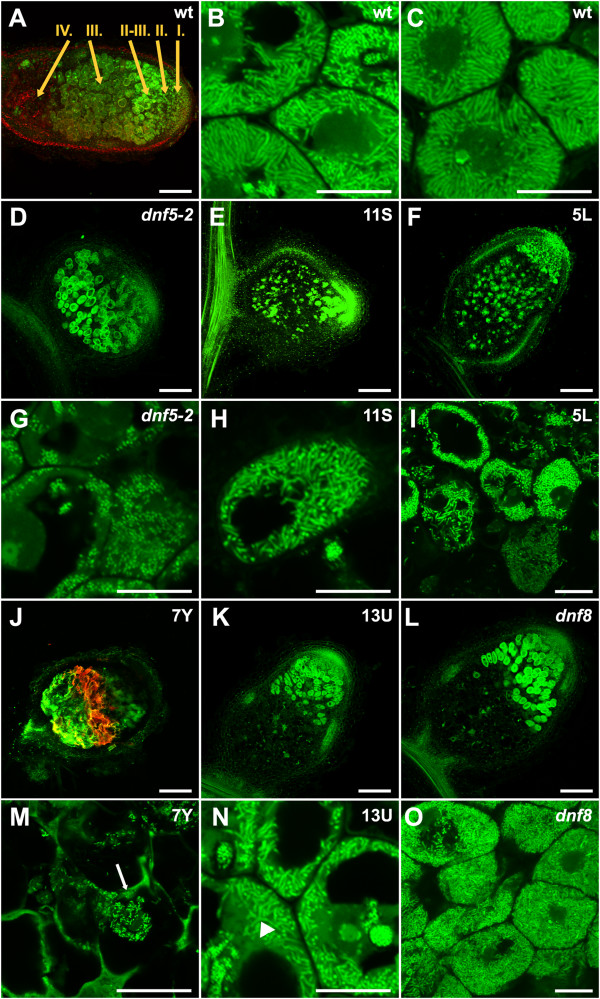
**Rhizobial differentiation in Fix- mutants.** Rhizobial colonization and bacterial morphology in *M. truncatula* wild-type **(A-C)** and Fix- mutant nodules **(D-O)**. Longitudinal sections of nodules 4 wpi with *S. meliloti* were stained with SYTO13 and analyzed by confocal microscopy. The regions of the mature wild-type (wt) nodules **(A)** indicated similarly as in Figure 2; but also with IV., senescence zone. Higher magnification revealed elongated bacteroids in the infected cells of the interzone and the nitrogen-fixation zone of wild type nodules **(B** and **C)**. The reduced zonation phenotype of *dnf5-2***(D)** is coupled with the lack of bacterial elongation **(G)**. The bacterial development is initiated in the nodules of the other ineffective mutants (5L, **E** and **H**; 11S, **F** and **I**; 7Y, **J** and **M**; *13U*, **K** and **N**; *dnf8*, **L** and **O**). Strong autofluorescence was observed in the proximal part of zone III of 7Y nodules **(J)**. Violet-laser excited (405 nm) autofluorescence was detected between 545–645 nm and pseudocolored in red **(A** and **J)**. Arrow shows an infection thread in panel **M**, arrowhead indicates branched bacteroids in panel **N**. Bars represent 200 μm in **A, D, E, F, J, K** and **L**, 20 μm in panels **B, C, G, H, I, M, N** and **O**.

The effective functioning of the symbiotic nodules is accompanied by the morphological differentiation of both the nodule cells and rhizobia [32]. To investigate the differentiation of rhizobia in the Fix- mutants, the bacterial morphology was analyzed following staining with the nucleic acid-binding dye SYTO13 and observed using confocal laser scanning microscopy [33]. Nodules of wild type plants showed characteristic bacteroid differentiation (Figure 4A-C), with the cells in the interzone and nitrogen fixation zone fully occupied by elongated bacteria (Figure 4B and C). The nodules of *dnf5-2* contained non-elongated rod shaped bacteria indicating the failure of bacteroid differentiation (Figure 4D and G). The 5L and 11S nodules are dotted with a few cells containing enlarged bacteria suggesting bacteroid differentiation limited to these cells (Figure 4E, F, H and I). 13U and *dnf8* nodules did not contain infected cells in the nitrogen fixation zone (Figure 4K, L, N and O), but elongated and branched bacteroids could be detected in the interzone cells. The few invaded cells of 7Y nodules contained slightly elongated bacteria (Figure 4J and M). The 7Y nodules showed strong autofluorescence (Figure 4J) that is likely associated with the brown pigmentation described earlier.

### The expression pattern of symbiotic marker genes differentiate the Fix- mutants

Nodule formation and the initiation of nitrogen fixation is the result of a complex developmental program accompanied by transcriptional changes in both symbiotic partners [32,34]. Several differentially expressed late nodulin genes can be used as markers to dissect the *M. truncatula*-rhizobial symbiotic interaction [29,32,34,35]. We selected ten symbiosis specific genes (*LEC4*, *Lb1*, *CAM1*, *N31*, *CP2*, *IPD3*, *NOD25*, *NOD26*, *NAP2* and *NCR121*) whose transcriptional profile [36] is presented in Additional file 4. We monitored rhizobia-induced expression of these symbiotic marker genes 14 days following bacterial inoculation (dpi) using quantitative RT-PCR (Additional file 5, Table 1).

**Table 1:** **Expression of symbiotic marker genes induced by *S. meliloti *in *M. truncatula *Fix- mutants**

***M. truncatula *line**	**Genes assayed for expression**									
	***MtlPD3***	***MtlEC4***	***MtCAM1***	***MtNOD26***	***MtCP***	***MtLb1***	***MtNCR121***	***MtNOD25***	***MtNAP2***	***Mtn31***
wt	**+**	**+**	**+**	**+**	**+**	**+**	**+**	**+**	**+**	**+**
*ipd3-1*	**+**	-	**(+)**	**(+)**	-	-	-	-	-	-
*dnf5-2*	**+**	**(+)**	-	**(+)**	-	-	**+**	-	-	-
5L	**+**	**+**	**+**	**+**	**+**	**(+)**	**+**	**+**	**+**	**(+)**
11S	**+**	**+**	**+**	**+**	**+**	**(+)**	**+**	**+**	**(+)**	-
*dnf7-2*	**+**	**+**	**+**	**(+)**	**+**	**+**	**+**	**+**	**(+)**	-
7Y	**+**	**+**	**+**	**(+)**	**+**	**(+)**	-	**(+)**	**+**	**(+)**
13U	**+**	**+**	**+**	**(+)**	**(+)**	**+**	-	**(+)**	**+**	-
*dnf8*	**+**	**+**	**+**	**+**	**+**	**(+)**	**+**	**+**	-	-

-, tested gene was not induced or negligible induction was detected (relative expression to wild-type was between 0.0 – 0.019); (+), reduced induction relative to wild-type (0.02 – 0.19); +, similar induction to wild-type (≥ 0.2).

Corresponding to the early arrest of the symbiotic process in *ipd3-1*, *dnf5-2* and 7Y (Figure 2), transcriptional activation of all the symbiotic marker genes were blocked or severely reduced in these mutants. One interesting exception however was *NCR121* which was induced in *dnf5-2* to levels comparable to wild-type, but which was strongly reduced in *ipd3-1* and 7Y. The expression of *MtNAP2*, encoding a nodule specific protein with unknown function was reduced in all ineffective mutants compared to wild-type plants. *MtNAP2* was expressed at lower level in nodules containing some infected cells in the nitrogen fixation zone but the complete failure of its induction was detected in *ipd3-1*, *dnf5-2*, *dnf8* and *dnf7-2* is related to the absence of nodule zonation or lack of bacterial occupancy in the nitrogen fixation zone (Figure 3). The *MtLB1*, *MtNOD25* and *MtNOD26* late nodulins were expressed at lower level in the ineffective mutants of this study than in wild type and they were not or hardly induced in the early ineffective mutants *ipd3-1* and *dnf5-2*. One of the marker genes of early senescence in nodules, the cysteine protease gene *MtCP* was expressed differently in the ineffective symbiotic mutants but the comparable level of expression with wild-type plants could be detected only in *dnf7-2* nodules. The *MtN31* transcript, encoding a nodule-specific Cys-rich protein (*NCR158*) could be detected at very low level in all ineffective mutants compared to wild-type, indicating that its expression is accompanied with the functioning symbiotic nodules. The expression data of the symbiotic marker genes were consistent with the block of the symbiotic nodule development detected by microscopic analysis.

## Discussion

The genetic dissection of symbiotic nitrogen fixation has identified several essential components of the symbiosis signaling pathway [11,12] and revealed some genes required for nodule function and bacterial infection [18-23,37,38]. In this paper we describe additional genetic dissection, particularly of the later stages of nodule function. This genetic screen revealed several new alleles of already defined genetic loci (*dmi2*, *dmi3*, *nsp2, nin*, *lin, dnf5* and *dnf7*; [18,29,39-43], two novel complementation groups (*dnf8* and *sta1*; [27]) and possibly additional 3 novel loci. The identification of a relatively high number of known symbiotic alleles either indicates the near-saturation of the symbiosis signaling pathway or a bias in the preference of the mutatable symbiotic loci in *M. truncatula*. It is possible that the similar growth conditions used in the different genetic screens may limit the tractable symbiotic loci and applying more precise or subtle conditions may allow the identification of additional symbiotic loci. Nevertheless, the identification of new genetic loci, particularly associated with the later stages of this process, indicates the genetic screens for nodulation mutants so far undertaken in *M. truncatula* are far from saturating. However, the combined action of a number of recent genetic screens in *M. truncatula* ([44] and http://medicago-mutant.noble.org/mutant/FNB.php)*,* may be taking basic nodulation screens towards genetic saturation.

Rhizobial colonization of nodule cells and formation of functioning symbiotic nodules involves simultaneous and coordinated development of the bacteria and the plant. Bacteria are released from the infection threads, enter into the cytoplasm via endocytosis and differentiate into bacteroids [2]. Small cysteine-rich peptides generated in nodule cells, but delivered to the released bacteria, are essential for bacteroid differentiation [20,45]. Appropriate delivery of nutrients is also presumed to be required for bacteroid maintenance and the delivery of sulphur has been shown to be essential at this step [23]. Many additional functions for the nodule are likely to be defined once the loci identified here and elsewhere [29,44,46-48], http://medicago-mutant.noble.org/mutant/FNB.php) have been cloned.

The inoculation of the ineffective mutants with rhizobia expressing *LacZ* reporter gene allowed us to compare the nodule phenotype of the new alleles of *dnf5* and *dnf7* to the previously identified ones [29]. Although nodules of different age were analyzed in this and the previous study [49], we found that *dnf5-2* showed similar defects in development of nodule zones as it was observed in *dnf5-1* nodules [49]. The sections of 10-day-old nodules of *dnf7-1* revealed that nodule cells harbored rhizobia in the infection and the intermediate zones [49], similarly as it was found in *dnf7-2* nodules.

Characterization of the mutant phenotypes and the analysis of gene expression allowed us to define the functional hierarchy of the impaired genes we identified. *IPD3* and *DNF5* appear to be the earliest acting genes, with both severe nodulation defects and greatly reduced symbiotic gene expression. However, both the degree of bacterial invasion and the expression of *NCR121* suggest that *DNF5* may act later in the process than *IPD3.* The disintegration of rhizobia and the sporadic brown pigmentation in the fixation zones of 5L/11S and 13U mutants show incompatible bacterial interactions and induction of early senescence. The genes impaired in 5L/11S and 13U appear to be required for the maintenance of bacterial infection, particularly in the nitrogen-fixation zone of the nodule. While *DNF8* has a similar nodulation defect, the gene profiling suggests that it acts at a slightly later stage than 5L/11S and 13U. The presence of bacteria in the early developmental zones of *dnf8* nodules and the complete lack of rhizobia in the nitrogen fixation zone probably indicates the arrest of rhizobial differentiation in this mutant. The strong brown pigmentation in the 7Y mutant may indicate misregulation of plant defenses. If this is correct then positioning 7Y within this sequence of loci is questionable, since the timing of the gene function may not be directly related to the stage at which the mutant aborts. However, such a statement could be true for all the genetic loci described and a role for *IPD3* (*CYCLOPS*) during symbiotic signaling [12,50,51] belies its apparently late mutant phenotype [30,52].

The characterization of the ineffective mutants may suggest the possible function of the impaired genes but in order to assess their actual function and characterize their gene products, these genes need to be cloned. Despite the great advantages of the insertion mutants that allow the recovery of the genomic sequences adjacent to the integration sites of the transposons [44], the genomic resources of *M. truncatula* also renders the rapid identification of deleted genes by either map-based cloning or transcript-based cloning, as successfully demonstrated in several recent papers [30,38]. The cloning and molecular characterization of genes defective in the ineffective *M. truncatula* mutants of this study will provide more details to the process of nodule invasion and differentiation and contribute to a better understanding of the molecular, developmental and differentiation events that support a nitrogen-fixing nodule.

## Conclusion

Plant mutants have been widely used for several years to identify genes required for nitrogen fixing symbiotic interaction between rhizobia and legumes. Here we describe a symbiotic mutant screen of fast neutron and EMS mutagenized *Medicago truncatula* plants carried out to identify additional symbiotic mutants. The identification of new alleles of known symbiotic genes and novel *Medicago truncatula* symbiotic mutants showing defects in nodule development and function indicated that symbiotic screens are far from saturation. The detailed characterization of the ineffective mutants allowed us to place the impaired *FIX* genes in a functional hierarchy which enables the selection of ineffective mutants impaired in a certain different stage of the symbiotic interaction for further analysis. The molecular identification of these *FIX* genes would elucidate their function and role in the symbiotic interaction. The collection of the ineffective nitrogen fixing mutants identified in this study can be the resource for identification of these new symbiotic genes.

## Methods

### Plant material, growth conditions and bacterial strains

The large-scale symbiotic screen was carried out on fast neutron (FN) bombarded and ethyl methanesulfonate (EMS) mutagenized seed populations of *M. truncatula* Jemalong line (FN) and genotype A17 of *M. truncatula* (EMS). The seeds of Jemalong genotype of *M. truncatula* line were exposed to fast neutron radiation as described by [25]. As described earlier [26], altogether 600 M2 pools (10–25 families/pool and 60 seedlings/family) of fast neuron radiated and EMS mutagenized pools were subjected to phenotypic screen.

For the symbiosis phenotypic screens, the nodulation tests and expression studies the *M. truncatula* seeds were scarified, sterilized, washed and imbibed as described in the *Medicago truncatula* handbook (http://www.noble.org/MedicagoHandbook). Sterilized seeds were vernalized for 5–7 days at 4°C and thereafter germinated on inverted agar (0.8% water agar) plates in dark at room temperature. For the symbiotic screen, mutagenized and control wild type seedlings were grown in 1:1 mixture of Terragreen and sand as described by [26]. Four-day-old *M. truncatula* seedlings were inoculated with *S. meliloti* strain B1 and plants were evaluated for their symbiotic phenotype 5–6 weeks postinoculation. Confirmation screening experiments were carried out in the same growing substrate in walk-in growth chambers as desribed by [26].

For phenotypic characterization, non-nodulating and ineffective nodulation mutants and wild-type plants were grown on either square plates containing buffered nodulation media (BNM) supplemented with 0.1 μM L-α-(2-aminoethoxyvinyl)-Gly (AVG) (Sigma-Aldrich, St. Louis) or in trays containing 3:1 mixture of zeolite substrate (Geoproduct Kft., Mád, Hungary) and sand. Four-day-old *M. truncatula* seedlings were inoculated with *S. meliloti* strain 1021 carrying the *hemA::lacZ* reporter construct (pXLGD4; [31]). *S. meliloti* culture was grown in liquid TA and at the log phase of the growth curve bacteria were pelleted and resuspended in liquid BNM. The final dilution 1:50 (OD_600_ 0.03-0.1) bacterial suspension was used for inoculation by flooding the roots on plate or adding 500 μl suspension to each plant in the tray. Plants on plates and trays were grown in growth chambers using the same light and temperature conditions as described earlier by [30].

Genetic crossings between symbiotic mutants and genotypes were carried out according to the method describe by [53]. To identify the map position of the symbiotic loci impaired in the ineffective symbiotic mutants, mutant plants were crossed to *M. truncatula* A20 genotype and F2 segregating populations were developed. The map positions were identified by analyzing the genotypes of the mapping populations for a genetic marker set of the *M. truncatula* genome [54,55].

### Microscopic analysis

For microscopic analysis, nodules were harvested 3 weeks postinoculation with *S. meliloti* 1021 (pXLGD4) and fixed with 4% formaldehyde in 1xPBS (pH 7.4) for 30 min on ice and rinsed 3×15 min in 1xPBS (pH 7.4). The nodules were embedded in 5% agarose (SeaKem® LE Agarose, Lonza) and 70 μm thick longitudinally sections were prepared with MICROM HM 650V Vibrotom. To visualize the β-galactosidase activity, sections were incubated in a staining solution containing 50–50 mM potassium ferricyanide and potassium ferrocyanide and 0,08% X-Gal (Fermentas, Lithuania) in 1xPBS (pH 7.4) for 30 min at room temperature. Stained nodule sections were observed under an Olympus BX41M microscope with ×10 ×20 objectives and images were captured using an Olympus Camedia E-10 digital camera.

To analyze the bacteroid morphology, nodule sections were stained in 1×PBS (pH 7.4) containing 5 μM SYTO13 (Invitrogen, Eugene, Oregon) for 20 min and rinsed with 1×PBS.

Confocal laser scanning microscopy was performed using Olympus Fluoview FV1000 confocal laser scanning microscope (Olympus Life Science Europa GmbH, Hamburg, Germany). Microscope configuration was the following: objective lenses: UPLSAPO 10× (dry, NA:0.4), UPLFLN 40× (oil, NA:1.3) and UPLSAPO 60x (oil, NA:1.35); sampling speed: 4μs/pixel; line averaging: 2×; scanning mode: sequential unidirectional; excitation: 488 nm (SYTO13); laser transmissivity: 5%; main dichroic beamsplitter: DM405/488; intermediate dichroic beamsplitter: SDM 490; emission filter: 505–530 nm. To capture autofluorescence (Figure 4A and J), 405 nm laser was used at 10% transmissivity and the spectral detector was set to 545–645 nm range.

### Acetylene reduction assay

Nitrogenase activity has been tested by acetylene reduction assay (ARA) with gas chromatograph GC-14B Shimadzu. Nodulated root sections were harvested 18 d after inoculation from mutant and wild-type plants grown on buffered nodulation media (BNM) plates as described above. Three to six nodules were placed into 3 ml glass vials sealed with rubber cap and immediately 0.15 ml acetylene were injected into the vials to incubate nodules in the presence of 10% (v/v) acetylene. Following 2 h incubation 500 μl samples and ethylene standards were injected into the gas chromatograph to determine the amount of produced ethylene. The nitrogenase activity was calculated as ARA units (nanomoles of ethylene per hour per nodule number). The ARA was carried out with three biological replicates.

### Gene expression analyses

For quantitative RT-PCR experiments *M. truncatula* plants were grown on plates using the same conditions as described for microscopic analysis. Medicago roots or nodulated roots were harvested 14 days postinoculation and total RNA was extracted by TRI Reagent (Sigma, USA) following the manufacturer’s protocol. The RNA samples were treated with RQ1 DNase I (Promega, USA) according to the manufacturer’s instructions. RNA were cleaned up by RNeasy Mini Kit (QIAGEN, DE), then genomic DNA free total RNA was quantified using a Nanodrop-1000 spectrophotometer (NanoDrop Technologies, USA) and checked for quality by gel electrophoresis.

Complementary DNA was prepared from 1 μg total RNA with SuperScript III First-Strand Synthesis System for RT-PCR (Invitrogen, USA) using oligo-dT primers according to the manufacturer’s instructions. Quantitative real time RT-PCR was performed on MiniOpticon™ System (BIO-RAD, USA) using Bio-Rad CFX Manager software 3,0. Maxima™ SYBR Green Master Mix (Thermo, USA) was used to monitor double-stranded (ds) DNA synthesis in 48-well plates. The final primer concentration of each gene-specific primer was 100 nM. The PCR conditions were as follows: a single cycle of 94°C for 10 min was followed by 45–50 cycles of 94°C for 15 sec, 58-60°C for 20 sec and 72°C for 20 sec. Following each PCR amplification, a melting curve was run to check genomic DNA contamination. To generate a melting profile, fluorescence of the samples is measured repeatedly as the temperature is gradually increased from 60°C to 95°C over 20 min after complete denaturation. Primer–dimer formation was estimated by running a control without template DNA. Results were expressed as a threshold cycle (C_T_) value, which were averaged from three replicate reactions. For normalization, C_T_ value of the reference gene was subtracted from the C_T_ value of the gene of interest (ΔC_T_). ΔΔC_T_ value was calculated by subtracting ΔC_T_ of the wild-type sample from the ΔC_T_ value of the different mutants. Fold induction (2^ΔΔCT^) of three independent experiments were averaged and plotted using SE.

Primer sequences used for qPCR are listed in Additional file 6. A gene (MTR_3g091440) with an ubiquitin domain was used as a reference gene and its intron sequence was utilized for checking genomic DNA contamination of cDNA samples as suggested by [56].

## Competing interest

The authors declare that they have no competing interests.

## Authors’ contributions

AD and BH equally contributed to this study. AD carried out gene expression analysis and phenotypic characterization. BH carried out phenotypic characterization, light microscopy analyses and produced plant materials for confocal imaging. JFM designed and managed the forward genetic screen. GH was involved in the genetic analysis of the mutant loci identified in this study. FA conducted confocal microscopic imaging. GEDO conceived and designed the mutant screen, discussed the results and edited the manuscript. PK participated in the mutant screen, carried out ARA assay, designed and managed the experiments of the mutant characterization and wrote the manuscript. All authors read and approved the final manuscript.

## Supplementary Material

Additional file 1 **Allelism tests between the mutants identified in this study and known symbiotic mutants of *M. truncatula *+ indicates that progeny displayed wild type symbiotic phenotypes.** - indicates that F1 hybrid plants did not form nodules (A) or displayed nitrogen deficiency symptoms under symbiotic conditions (B). Numbers in parenthesis represent the number of plants scored for symbiotic phenotype and the numbers of pods from which seeds originated. The allelic relationships are highlighted with grey. Click here for file

Additional file 2 **Segregation analysis for the ineffective symbiotic phenotype of the eight selected ineffective mutants isolated in the symbiotic mutant screen.** Segregation data presented for backcross and F2 segregation populations. Χ^2^ values were calculated based on the 3:1 segregation ratio; P >0.05; * at 0.025 significant level; ** at 0.01 significant level; *** at 0.005 significant level. Click here for file

Additional file 3 **The map position of 3 symbiotic loci 13U, 5L/11S and 7Y.** The map positions of the three symbiotic loci were determined using three F2 mapping populations containing 238, 288 and 81 individuals. The genetic markers used for mapping are indicated alongside the proportional bars representing each linkage groups of *M. truncatula*. The red vertical bars represent the region wherein the symbiotic loci could be located. The map positions of genetic markers (cM) are transferred from the *M. truncatula* genetic map developed by Mun et al. [55]. Click here for file

Additional file 4 **The expression profile of ten selected symbiotic marker genes generated based on data of *M. truncatula *Gene Expression Atlas** (http://mtgea.noble.org/v3/). Click here for file

Additional file 5 **Expression analysis of selected nodule specific genes in *M. truncatula *ineffective mutants 14 days after inoculation with *S. meliloti *1021.** The expression of genes *MtLEC4, MtLB, MtCAM1, MtN31, MtCP, MtIPD3, MtNOD25, MtNOD26, MtNAP2* and *MtNCR121* were analyzed relative to wild type using real-time RT-PCR. Three biological replicates for each mutant with three technical repeats were used for the analysis. A gene (MTR_3g091440), member of the ubiquitin protein family was used for data normalization as suggested by Kakar and co-workers [56]. Error bars represent SE. Click here for file

Additional file 6 Primers used in this study. Click here for file
