# Supplementary material for: The identification of novel loci required for appropriate nodule development in Medicago truncatula
Source: BMC Plant Biol. 2013 Oct 11;13:157. doi: 10.1186/1471-2229-13-157 (PMC3852326; doi:10.1186/1471-2229-13-157)
Supplement: Additional file 1 — Allelism tests between the mutants identified in this study and known symbiotic mutants of M. truncatula + indicates that progeny displayed wild type symbiotic phenotypes. - indicates that F1 hybrid plants did not form nodules (A) or displayed nitrogen deficiency symptoms under symbiotic conditions (B). Numbers in parenthesis represent the number of plants scored for symbiotic phenotype and the numbers of pods from which seeds originated. The allelic relationships are highlighted with grey. [file 1471-2229-13-157-S1.doc]

**Additional file 1** - Allelism tests between the mutants identified in this study and known symbiotic mutants of M. truncatula


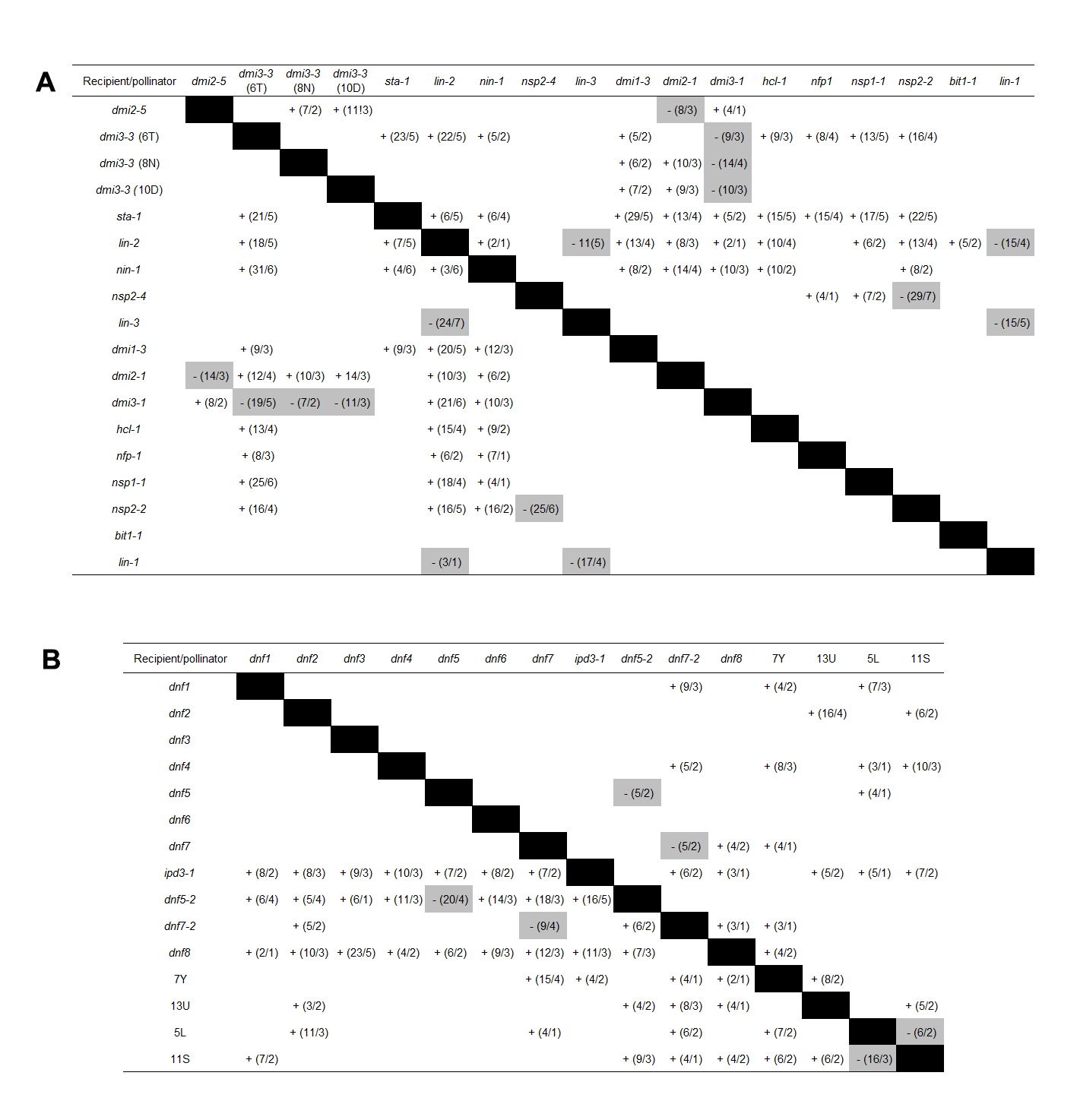


+ indicates that progeny displayed wild type symbiotic phenotypes. - indicates that F1 hybrid plants did not form nodules (A) or displayed nitrogen deficiency symptoms under symbiotic conditions (B). Numbers in parenthesis represent the number of plants scored for symbiotic phenotype and the numbers of pods from which seeds originated. The allelic relationships are highlighted with grey.
