# Supplementary material for: The identification of novel loci required for appropriate nodule development in Medicago truncatula
Source: BMC Plant Biol. 2013 Oct 11;13:157. doi: 10.1186/1471-2229-13-157 (PMC3852326; doi:10.1186/1471-2229-13-157)
Supplement: Additional file 2 — Segregation analysis for the ineffective symbiotic phenotype of the eight selected ineffective mutants isolated in the symbiotic mutant screen. Segregation data presented for backcross and F2 segregation populations. Χ2 values were calculated based on the 3:1 segregation ratio; P >0.05; * at 0.025 significant level; ** at 0.01 significant level; *** at 0.005 significant level. [file 1471-2229-13-157-S2.doc]

**Additional file 2 – Segregation ratio of ineffective phenotypes in backcrossed and A20-crossed segregation populations.**
