# Supplementary figures and images for: The identification of novel loci required for appropriate nodule development in Medicago truncatula
Source: BMC Plant Biol. 2013 Oct 11;13:157. doi: 10.1186/1471-2229-13-157 (PMC3852326; doi:10.1186/1471-2229-13-157)

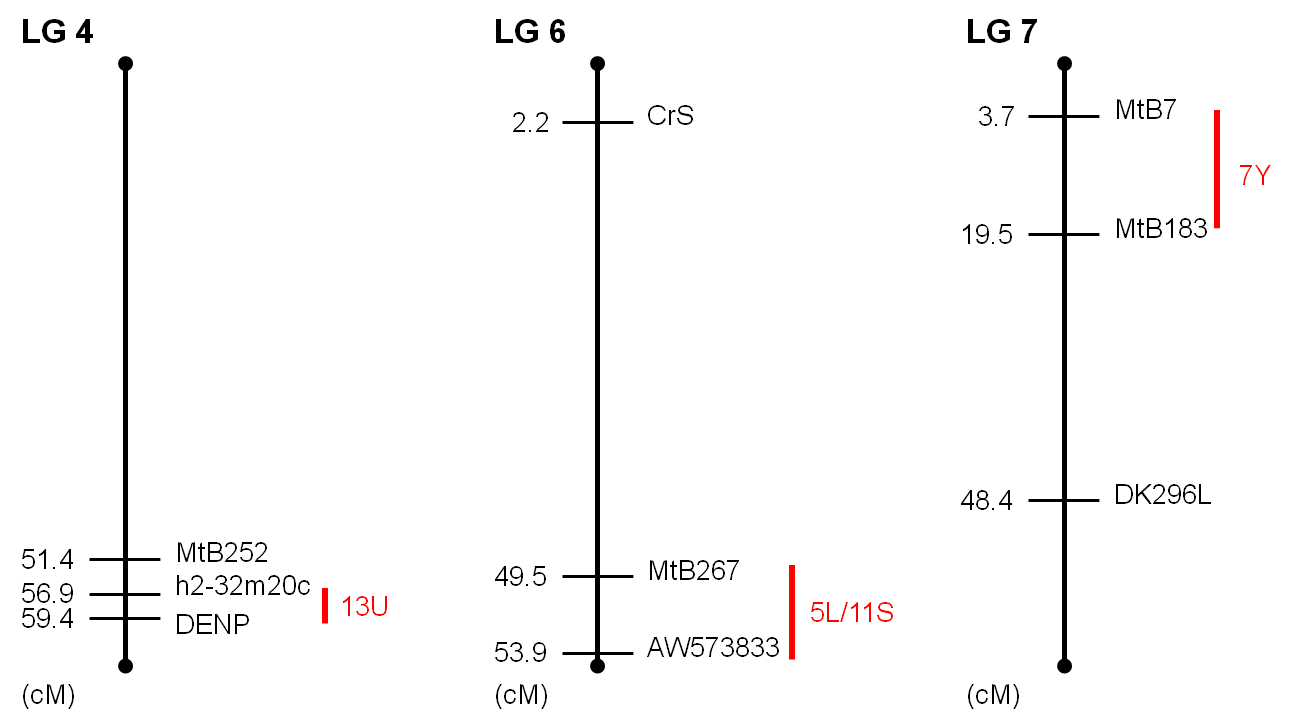

Supplement: Additional file 3 — The map position of 3 symbiotic loci 13U, 5L/11S and 7Y. The map positions of the three symbiotic loci were determined using three F2 mapping populations containing 238, 288 and 81 individuals. The genetic markers used for mapping are indicated alongside the proportional bars representing each linkage groups of M. truncatula. The red vertical bars represent the region wherein the symbiotic loci could be located. The map positions of genetic markers (cM) are transferred from the M. truncatula genetic map developed by Mun et al. [55]. [file 1471-2229-13-157-S3.tiff]

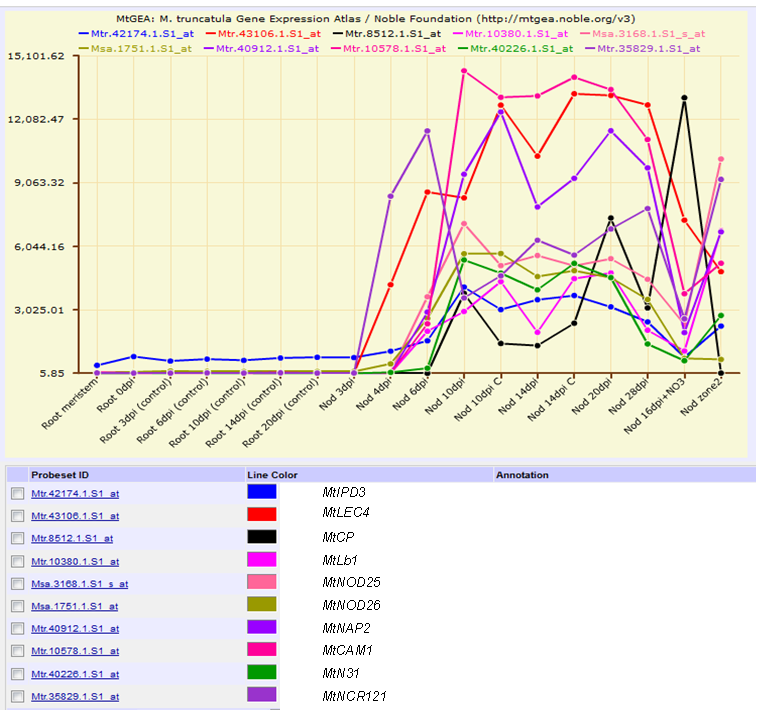

Supplement: Additional file 4 — The expression profile of ten selected symbiotic marker genes generated based on data of M. truncatula Gene Expression Atlas (http://mtgea.noble.org/v3/). [file 1471-2229-13-157-S4.tiff]
