# Supplementary material for: The identification of novel loci required for appropriate nodule development in Medicago truncatula
Source: BMC Plant Biol. 2013 Oct 11;13:157. doi: 10.1186/1471-2229-13-157 (PMC3852326; doi:10.1186/1471-2229-13-157)
Supplement: Additional file 5 — Expression analysis of selected nodule specific genes in M. truncatula ineffective mutants 14 days after inoculation with S. meliloti 1021. The expression of genes MtLEC4, MtLB, MtCAM1, MtN31, MtCP, MtIPD3, MtNOD25, MtNOD26, MtNAP2 and MtNCR121 were analyzed relative to wild type using real-time RT-PCR. Three biological replicates for each mutant with three technical repeats were used for the analysis. A gene (MTR_3g091440), member of the ubiquitin protein family was used for data normalization as suggested by Kakar and co-workers [56]. Error bars represent SE. [file 1471-2229-13-157-S5.doc]

**Additional file 5 - qRT-PCR of symbiotic marker genes**


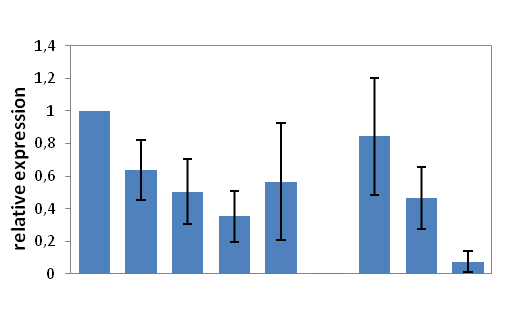

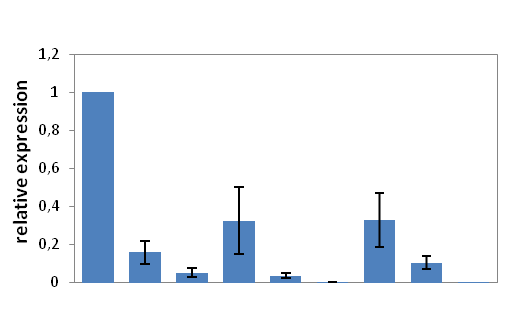

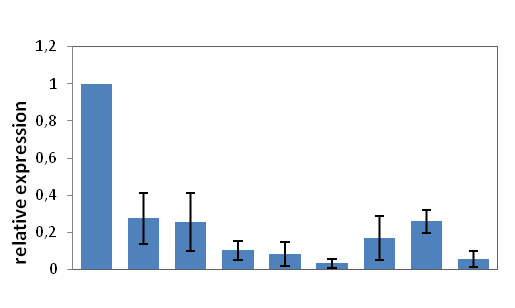

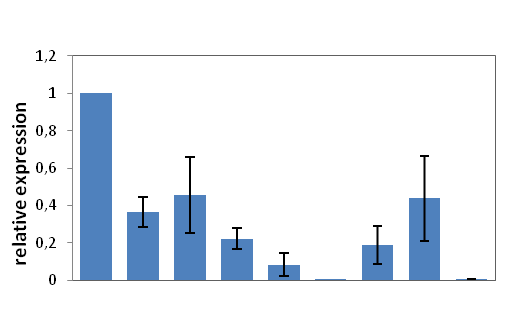


***MtNOD25***

***MtNOD26***


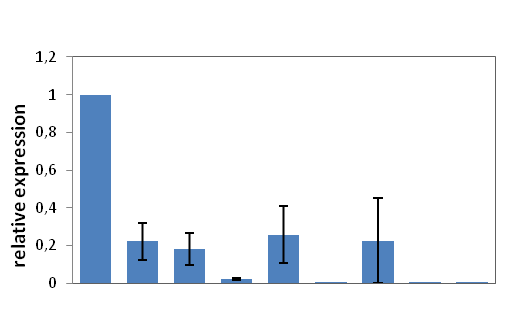

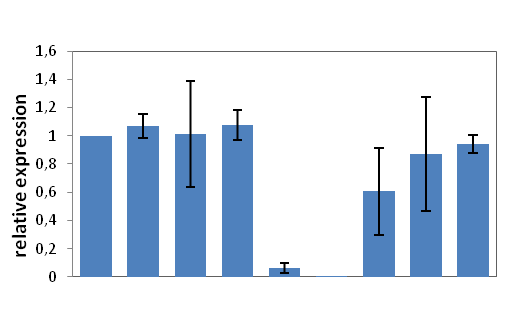

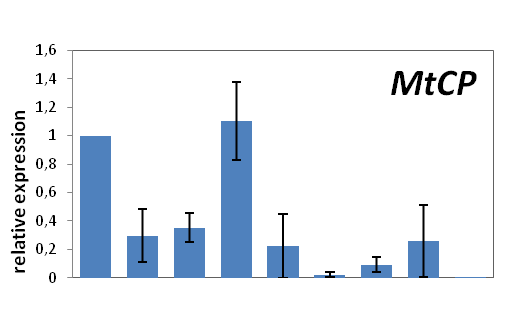

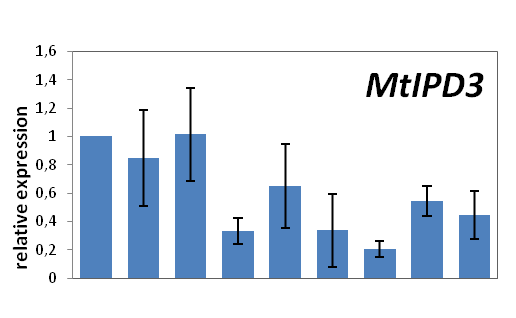

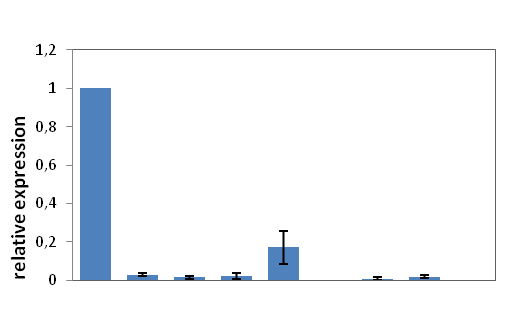

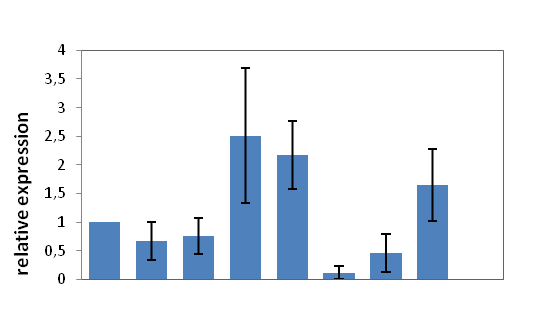


***MtCAM1***

**MtN31**


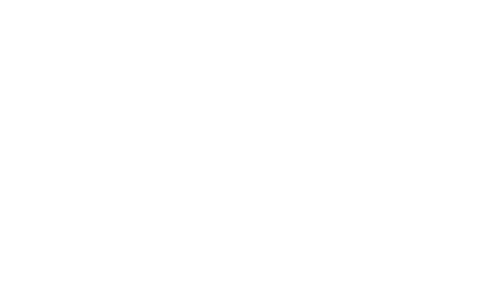


***MtLEC4***

***MtLB1***

***MtNAP2***

***MtNCR121***

**wt 5L 11S *dnf7-2* 7Y *ipd3-1* 13U *dnf8* *dnf5-2***

**wt 5L 11S *dnf7-2* 7Y *ipd3-1* 13U *dnf8* *dnf5-2***

**wt 5L 11S *dnf7-2* 7Y *ipd3-1* 13U *dnf8* *dnf5-2***

**wt 5L 11S *dnf7-2* 7Y *ipd3-1* 13U *dnf8* *dnf5-2***

**wt 5L 11S *dnf7-2* 7Y *ipd3-1* 13U *dnf8* *dnf5-2***

**wt 5L 11S *dnf7-2* 7Y *ipd3-1* 13U *dnf8* *dnf5-2***

**wt 5L 11S *dnf7-2* 7Y *ipd3-1* 13U *dnf8* *dnf5-2***

**wt 5L 11S *dnf7-2* 7Y *ipd3-1* 13U *dnf8* *dnf5-2***

**wt 5L 11S *dnf7-2* 7Y *ipd3-1* 13U *dnf8* *dnf5-2***

**wt 5L 11S *dnf7-2* 7Y *ipd3-1* 13U *dnf8* *dnf5-2***
