# Supplementary material for: The identification of novel loci required for appropriate nodule development in Medicago truncatula
Source: BMC Plant Biol. 2013 Oct 11;13:157. doi: 10.1186/1471-2229-13-157 (PMC3852326; doi:10.1186/1471-2229-13-157)
Supplement: Additional file 6 — Primers used in this study. [file 1471-2229-13-157-S6.doc]

**Additional file 6 – Primers used in this study.**
